# A genomic case study of desmoplastic small round cell tumor: comprehensive analysis reveals insights into potential therapeutic targets and development of a monitoring tool for a rare and aggressive disease

**DOI:** 10.1186/s40246-016-0092-0

**Published:** 2016-11-18

**Authors:** Elisa Napolitano Ferreira, Bruna Durães Figueiredo Barros, Jorge Estefano de Souza, Renan Valieris Almeida, Giovana Tardin Torrezan, Sheila Garcia, Ana Cristina Victorino Krepischi, Celso Abdon Lopes de Mello, Isabela Werneck da Cunha, Clóvis Antonio Lopes Pinto, Fernando Augusto Soares, Emmanuel Dias-Neto, Ademar Lopes, Sandro José de Souza, Dirce Maria Carraro

**Affiliations:** 1 International Research Center/CIPE, A.C. Camargo Cancer Center, São Paulo, SP Brazil; 2 Instituto Metrópole Digital, Federal University of Rio Grande do Norte, Natal, RN Brazil; 3 Institute of Biosciences, University of São Paulo, São Paulo, SP Brazil; 4 Departament of Abdominal Surgery, A.C. Camargo Cancer Center, São Paulo, SP Brazil; 5 Department of Anatomic Pathology, A.C. Camargo Cancer Center, São Paulo, SP Brazil; 6 Federal University of Rio Grande do Norte, Natal, RN Brazil

**Keywords:** Desmoplastic small round cell tumor, Genomic profiling, Whole-exome sequencing, EWS-WT1 gene fusion, Personalized biomarker, Liquid biopsy

## Abstract

**Background:**

Genome-wide profiling of rare tumors is crucial for improvement of diagnosis, treatment, and, consequently, achieving better outcomes. Desmoplastic small round cell tumor (DSRCT) is a rare type of sarcoma arising from mesenchymal cells of abdominal peritoneum that usually develops in male adolescents and young adults. A specific translocation, t(11;22)(p13;q12), resulting in *EWS* and *WT1* gene fusion is the only recurrent molecular hallmark and no other genetic factor has been associated to this aggressive tumor. Here, we present a comprehensive genomic profiling of one DSRCT affecting a 26-year-old male, who achieved an excellent outcome.

**Methods:**

We investigated somatic and germline variants through whole-exome sequencing using a family based approach and, by array CGH, we explored the occurrence of genomic imbalances. Additionally, we performed mate-paired whole-genome sequencing for defining the specific breakpoint of the EWS-WT1 translocation, allowing us to develop a personalized tumor marker for monitoring the patient by liquid biopsy.

**Results:**

We identified genetic variants leading to protein alterations including 12 somatic and 14 germline events (11 germline compound heterozygous mutations and 3 rare homozygous polymorphisms) affecting genes predominantly involved in mesenchymal cell differentiation pathways. Regarding copy number alterations (CNA) few events were detected, mainly restricted to gains in chromosomes 5 and 18 and losses at 11p, 13q, and 22q. The deletions at 11p and 22q indicated the presence of the classic translocation, t(11;22)(p13;q12). In addition, the mapping of the specific genomic breakpoint of the EWS-WT1 gene fusion allowed the design of a personalized biomarker for assessing circulating tumor DNA (ctDNA) in plasma during patient follow-up. This biomarker has been used in four post-treatment blood samples, 3 years after surgery, and no trace of EWS-WT1 gene fusion was detected, in accordance with imaging tests showing no evidence of disease and with the good general health status of the patient.

**Conclusions:**

Overall, our findings revealed genes with potential to be associated with risk assessment and tumorigenesis of this rare type of sarcoma. Additionally, we established a liquid biopsy approach for monitoring patient follow-up based on genomic information that can be similarly adopted for patients diagnosed with a rare tumor.

**Electronic supplementary material:**

The online version of this article (doi:10.1186/s40246-016-0092-0) contains supplementary material, which is available to authorized users.

## Background

Comprehensive molecular profiling is an especially important tool to gain insights on the biological pathways involved in tumor onset and to improve the management and treatment of rare tumors. Desmoplastic small round cell tumor (DSRCT) is a very rare type of sarcoma, with an age-adjusted incidence rate of 0.3 cases/million [1], which typically arises from the abdominal or pelvic peritoneum and occurs mainly in male adolescents and young adults (peak incidence at 20–24 years of age) [1]. Current therapeutic approaches involve the use of multimodal therapeutic regimen, including aggressive polychemotherapy, debulking surgery, and whole abdominal radiation [2].

A specific translocation, t(11;22)(p13;q12), is detected in DSRCT cases, juxtaposing the Ewing’s sarcoma gene (*EWSR1*) to the Wilm’s tumor gene (*WT1*). The chimeric transcript containing the 5′ region of the *EWSR1*, which includes the N-terminal transactivation domain of EWS, and the 3′ sequence of *WT1* containing 2–4 zinc finger domains have been shown to upregulate EGR-1 [3] and induce the expression of PDGFA [4] and IGF1R [5].

Apart from this translocation, no other recurrent genomic alteration has been reported in DSRCT cases. Silva et al. [6] detected a somatic amplification involving *AURKB* and *MCL1* genes in one patient, and La Starza et al. [7] found specific genomic imbalances, including gain at chromosome 3 reported in two cases and chromosome 5 polysomy in one case. In terms of point mutations, the data is even scarcer. Variants of unknown clinical significance were reported in *ARID1A* and *RUNX1* genes in one patient [6], whereas in another study, no mutations were detected in a panel of 29 genes evaluated in a cohort of 24 DSRCT cases [8]. This limited genomic information about DSRCT impairs new and more efficient therapeutic opportunities for the young patients affected with this rare tumor.

Here, aiming to contribute with the knowledge of the genomic abnormalities that underlies DSRCT, we performed a comprehensive genomic profiling using a family based approach in one case of DSRCT diagnosed in a young male patient with pelvic tumor, who presented excellent outcome sustained for above 3 years. We identified somatic mutations in a genomic background of rare germline variants either homozygous or as compound heterozygous inheritance, which can improve the understanding of the genetic basis of this rare tumor. We have also generated the profile of genomic imbalances, which was confirmed by whole-exome sequencing. In addition, as we were able to define the precise genomic breakpoints of the EWS-WT1 translocation by whole-genome and Sanger sequencing, we managed to establish a personalized strategy for tracking DNA tumor traces in plasma, allowing an accurate monitoring of tumor recurrence.

Overall, our analysis revealed potential genes and pathways associated with this rare sarcoma and demonstrated the feasibility of using genomic profiling for the benefit of patients affected by rare tumors by developing a personalized monitoring strategy.

## Methods

### DNA extraction

Tumor tissues and blood samples were collected following the technical and ethical procedures of A.C. Camargo Tumor Bank, registered at National Council for Ethics in Research by the number B001 [9]. Genomic DNA and plasma DNA were extracted in DNA and RNA Bank [10] using QIASymphony DNA Mini kit (QIAGEN, Hilden, Germany) for tumor and leukocyte DNA and QIAamp DNA blood Midi kit (QIAGEN, Hilden, Germany) for plasma DNA, following standard procedures.

### Target sequencing

Target sequencing was performed using the Ion AmpliSeq™ Comprehensive Cancer Panel, which comprises all exons from 409 genes associated with different types of tumors (AmpliSeq, Ion Torrent™). This panel, based on multiplex PCR, was performed with as little as 40 ng of DNA from the tumor sample. Library was prepared based on Ion AmpliSeq™ Library Preparation protocol and sequenced at Ion Proton™ platform (Ion Torrent™), according to the manufacturer’s instructions.

### Whole-exome sequencing

Whole-exome sequencing of the tumor and leukocyte DNA samples from the patient and his mother were performed using the TargetSeq™ Exome Enrichment Kit (Life Technologies), followed by paired-end sequencing (75 × 50) in SOLiD 5500xl System (Life Technologies). Leukocyte DNA sample from his father was submitted for whole-exome sequencing using Ion Xpress™ Plus Fragment Library kit and Ion TargetSeq™ Exome Enrichment Kit (Life Technologies), followed by sequencing at Ion Proton™ platform (Ion Torrent™), according to the manufacturer’s instructions. Single-end sequencing was performed on an Ion PI™ Chip v2 with 200 pb sequencing kit (Ion Torrent™).

### Whole-genome sequencing

Whole-genome sequencing was performed by mate-paired DNA libraries prepared using the 5500 SOLiD™ Mate-Paired Library Construction Kit (Life Technologies), following the manufacturer’s instructions. Briefly, genomic DNA from tumor and leukocyte of the patient was sheared with a Covaris sonicator into approximately 2 Kb fragments, circularized with mate-paired adaptors, nick-translated and digested, incorporated with sequencing adaptors and individual barcodes (distinct barcodes were used for the tumor and leukocyte DNA), and submitted to emulsion PCR. Mate-paired sequencing (60 × 60) was performed in a SOLiD 5500xl System (Life Technologies).

### Sanger sequencing

For validation, primers were designed flanking the variants, in order to generate fragments of nearly 400 bp. PCR reactions were performed using GoTaq® Green Master Mix (Promega, Madison, WI, USA), using 15 ng of DNA, with 300 nM of each primer, for a final reaction volume of 20 μL. Approximately 200 ng of PCR-amplified fragments were purified with ExoSAP-IT (USB Corporation, Cleveland, OH, USA) and sequenced in both directions. All alterations were evaluated in all four samples (mother, father, and patient’s leukocyte and tumor). Products were analyzed using an ABI 3130xl DNA sequencer (Applied Biosystems, Foster City, CA, USA), and sequences were aligned with the respective gene reference sequence using CLC Genomics Workbench Software (QIAGEN, Hilden, Germany).

### Comparative genome hybridization based on microarrays (array CGH)

Comparative genomic hybridization based on microarrays was performed in a commercial whole-genome 180 K platform containing 180,000 oligonucleotide probes (Agilent Technologies; design 22060), using DNA from the tumor sample. Reference DNA was a commercially available human pool of samples from multiple anonymous healthy donors (Promega Corporation). Technical procedures are described in Torrezan et al. [11]. Hybridization and washing were performed as recommended by the manufacturer. Scanned images were processed using Feature Extraction 10.7.3.1 software (Agilent Technologies), and array CGH analysis was conducted with Nexus Copy Number software 7.0 (Biodiscovery). We used the FASST2 segmentation algorithm, according to the following settings: minimum of five consecutive probes (effective resolution of ~70 Kb for CNA calling), significance threshold set at 10^−8^, and threshold log_2_ Cy3/Cy5 of 0.33 and −0.3 for gains for loss, respectively, and 1.2 and −1.1 for high copy number gains and homozygous losses, respectively. All copy number alterations are reported in the Database of Genomic Variants [12].

### Bioinformatics analysis

#### Comprehensive cancer panel

Sequencing reads from Ion Proton™ were mapped to the reference genome (GRCh37/hg19) with TMAP (torrent mapper 4.2.18). Sequence variants (SNVs and indels) were identified with Torrent Variant Caller 4.0-5, followed by confirmation by GATK protocol vs3.2-2-gec30cee [13]. Variants were annotated using SnpEff version 3.5d (build 2014-03-05) [14].

#### Whole-exome sequencing

Sequencing reads from SOLiD 5500xl System were mapped to the reference genome (GRCh37/hg19) with Lifescope (LifeScope™ Genomic Analysis Software v2.5.1). Sequencing reads from Ion Proton™ were mapped to the reference genome (GRCh37/hg19) with TMAP (torrent mapper 4.2.18). Sequence variants (SNVs and indels) were identified following the GATK protocol vs3.2-2-gec30cee [13] for SOLiD 5500XL System and with Torrent Variant Caller 4.0-5, followed by GATK protocol vs3.2-2-gec30cee for Ion Proton reads. Variants were annotated using SnpEff version 3.5d (build 2014-03-05) [14] and an in-house developed script. Identified variants were compared to dbNSFP version 2.4 [15, 16]; COSMIC v69 [17]; 1000 Genomes [18]; NHLBI GO Exome Sequencing Project version ESP6500SI-V2 [19]; HapMap [20]; and dbSNP version 138 [21, 22] for further annotation. Somatic variants were defined for regions with a minimum coverage of 10× for both tumor and leukocyte sample from the patient and minimum variant frequency of 20% in the tumor only. De novo variants were defined with a minimum coverage of 10× for leukocyte samples of the patient and his parents and with a minimum variant frequency of 30%. To identify rare polymorphisms inherited in homozygosity, we selected variants with a minimum coverage of 10× for leukocyte samples of the patient and his parents, detected in heterozygosis in both parents and homozygosis in the patient presenting a minor allele frequency ≤10% in the public databases (1000 Genome [18], NHLBI GO Exome Sequencing Project [19], and HapMap [20]). To identify germline compound heterozygosis cases, we identified genes with two distinct heterozygous mutations in the patient, where each variant was exclusively present in one of his parents in heterozygosity and detected in regions with a minimum coverage of 10× for all leukocyte samples. We discarded variants that are detected with a minor allele frequency above 10% in the 1000 Genome Project [18]. Copy number alterations were detected using the bioinformatics packages Excavator (version 2.2) [23] and cn.mops (version 1.8.9) [24] by comparing exome data from the tumor to leukocyte from the patient. For visualization, we used circos 0.67-7 package [25].

#### Whole-genome sequencing

For detecting structural variations, mate-pair reads obtained by SOLiD 5500xl System were analyzed by svdetect (version r0.8b) [26].

#### Ingenuity Pathway Analysis

We applied the core analysis of Ingenuity Pathway Analysis (IPA) system (QIAGEN, Germantown, MD, USA) to identify gene interaction networks.

### Digital droplet PCR

Digital droplet PCR assays were carried out using the QX200™ Droplet Digital™ (ddPCR™) System (Bio-Rad). A primer-probe assay labeled with FAM was designed for the amplification of wild-type *WT1* gene and a primer-probe assay labeled with HEX was designed for the amplification of the gene fusion event (EWS-WT1). For the amplification reaction, we used 1× ddPCR Supermix, 1× primer-probe assay (FAM), 1× primer-probe assay (HEX), and 4 μl of DNA. Droplet generation, PCR amplification, and droplet counting were performed following the manufacturer’s recommendations.

DNA samples from tumor tissue (60 ng–6 pg) and from leukocytes (6 ng) were used as positive and negative controls, respectively. We loaded 4 μl of 30 μl (1/8) of cfDNA samples from the patient. We also used DNA and cell-free DNA extracted from leukocytes and plasma, respectively, from healthy donors as negative controls and performed non-template control. All reactions were performed with at least two replicates.

## Results

In this study, we performed a comprehensive genomic profiling of one case of desmoplastic small round cell tumor (DSRCT) by a combination of targeted sequencing, array CGH, whole genome, and whole exome applied in a family based format. The patient studied here is a 26-year-old male, who presented at A.C. Camargo Cancer Center in November, 2011, with a large abdominal mass and a small nodule on the pelvic region. Staging images showed that the disease was limited to the abdominal cavity. He underwent a CT-guided biopsy that revealed a desmoplastic small round cell tumor (DSRCT), showing positivity for EMA, desmin, and nuclear staining for WT1 (carboxy-terminus antibody). FISH analysis was positive for EWS translocation.

The patient started systemic treatment with 4 cycles of vincristine, cyclophosphamide, and doxorubicin (VAC) and alternated with ifosfamide, carboplatin, and etoposide (ICE). After 4 cycles, the best response was stable disease, with minor reduction in the tumor dimensions. He underwent complete surgical cytoreduction, with resection of the large mass and resection of peritoneal implant on the pelvic region, and hyperthermic intraperitoneal chemotherapy (HIPEC), with cisplatin and doxorubicin. The patient presented a complete recovery from these procedures. After that, the patient received four more cycles of chemotherapy and total abdominal irradiation (total of 30 Gy). After a follow-up of 48 months since surgery, the patient is asymptomatic, with no signs of disease (Additional file 1: Figure S1).

The comprehensive genomic profiling was carried out in multiple fronts. To identify actionable mutations, we carried out targeted sequencing of the most important actionable genes in the tumor sample. Further, to identify genes and mutations possibly involved with tumor onset and predisposition, we performed whole exome sequencing, using the DSRCT tumor sample and blood samples from the patient and his both parents (Additional file 2: Table S1). Array CGH was performed in tumor sample to identify structural rearrangements and copy number imbalances. Whole-genome sequencing defined one tumor marker used to precisely monitor patient after treatment.

### Producing a portrait of somatically acquired variants

To identify actionable mutations or pathways related to DSRCT in this patient, we initially performed targeted sequencing using a cancer-oriented gene panel composed of 409 genes in the tumor sample (Comprehensive Cancer Panel – Thermo Scientific). Since no actionable mutation for targeted therapy was detected in the tumor, we investigated the complete landscape of somatic mutations by whole-exome sequencing (WES) the tumor and the patient’s leukocyte. The analysis of the tumor revealed 15 somatic acquired mutations, 12 of which were protein-affecting variants (validated by capillary sequencing) including one non-sense mutation in the *ZNF808* gene, one nucleotide change at the 3’ splice site of *RIMS4*, and 10 missense mutations considered disease associated by at least one pathogenicity prediction program (Table 1).

**Table 1:** Description of somatically acquired point mutations detected in the DSRCT by whole-exome sequencing

Chromosome position	Gene symbol	Variant description	Variant type	Frequency (tumor coverage)	Coverage of leukocyte DNA	dbSNP	PolyPhen	Sift	Mutation taster
chr3:436494	*CHL1*	c.3033A>G, p.A111A	Synonymous	35% (55×)	53×	–	–	–	–
chr6:134305546	*TBPL1*	c.315T>G, p.V105V	Synonymous	23% (31×)	28×	–	–	–	–
chr12:34179763	*ALG10*	c.1335A>T, p.A445A	Synonymous	43% (82×)	66×	–	–	–	–
chr1:45808899	*TOE1*	c.1058C>T, p.P353L	Missense	20% (15×)	18×	rs145913038	Benign	Damaging	Polymorphism
chr2:162875307	*DPP4*	c.1352C>T, p.P451L	Missense	37% (41×)	46×	–	Deleterious	Tolerated	Disease causing
chr5:126753368	*MEGF10*	c.1169G>C, p.G390C	Missense	22% (82×)	39×	–	Deleterious	Damaging	Disease causing
chr5:26915867	*CDH9*	c.394G>C, p.D132Y	Missense	25% (71×)	75×	–	Deleterious	–	Disease causing
chr6:123319098	*CLVS2*	c.176G>A, p.R59Q	Missense	40% (25×)	20×	–	Deleterious	Damaging	Disease causing
chr8:106813312	*ZFPM2*	c.1002T>A, p.S334R	Missense	28% (36×)	44×	–	Deleterious	Tolerated	Disease causing
chr8:72983969	*TRPA1*	c.245T>C, p.I82T	Missense	36% (45×)	35×	–	Deleterious	Damaging	Disease causing
chr15:37385900	*MEIS2*	c.521G>A, p.R86Q	Missense	26% (31×)	23×	–	Possible damaging	Damaging	Disease causing
chr16:76495948	*CNTNAP4*	c.1210G>T, p.A404S	Missense	33% (42×)	43×	–	Benign	Tolerated	Disease causing
chr17:10300120	*MYH8*	c.4362G>T, p.K1454N	Missense	26% (38×)	33×	–	Deleterious	–	Disease causing
chr19:53057457	*ZNF808*	c.1288G>T, p.E430Ter	Nonsense	43% (30×)	27×	–	–	Tolerated	Polymorphism
chr20:43385680	*RIMS4*	c.455-2T>A	3’ splice site	28% (29×)	37×	–	–	–	–

**Fig. 1:**
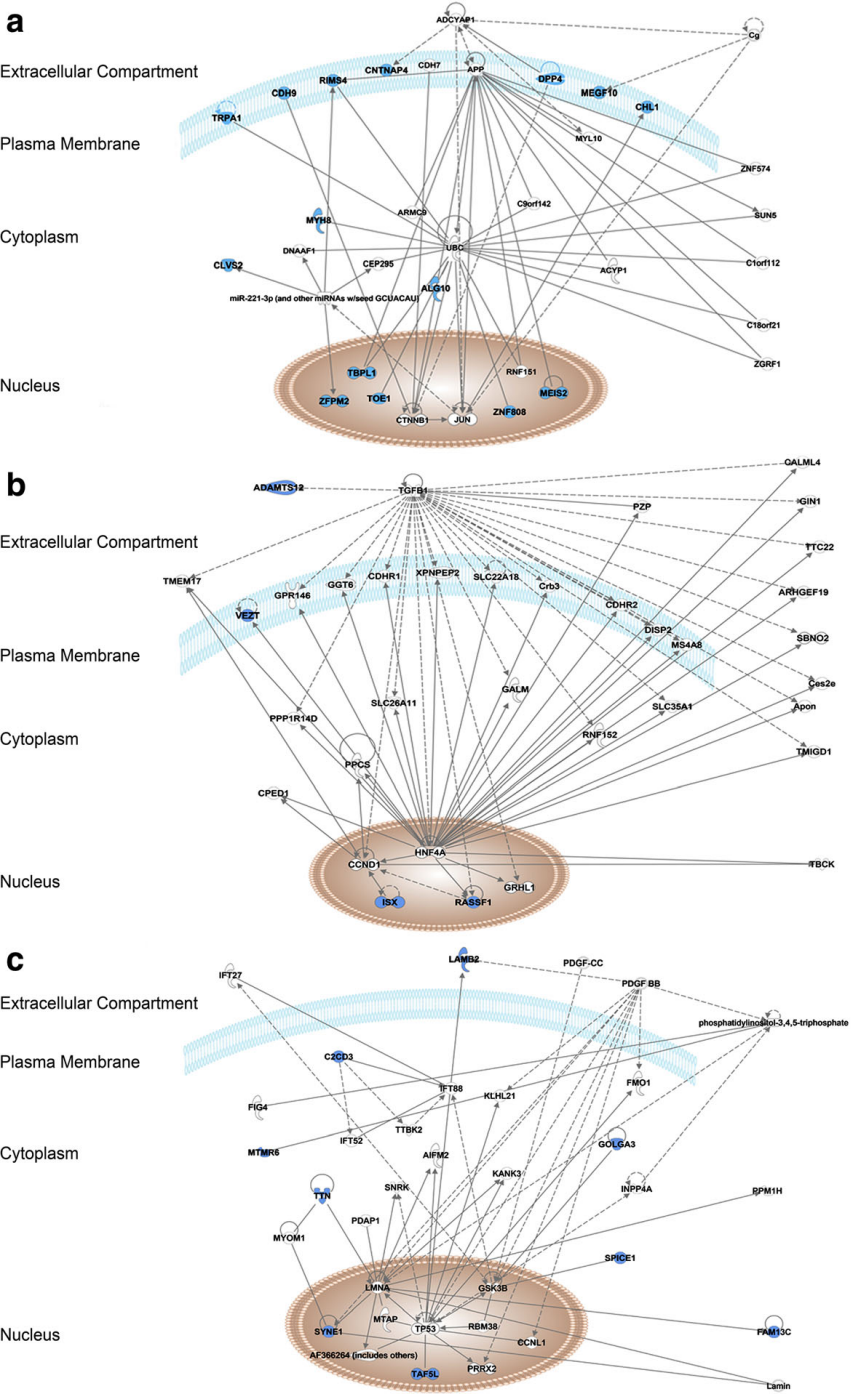
Network analysis by IPA. **a** Interaction network of genes harboring protein-affecting somatic mutations (network score = 45) is associated with the top disease and functions: cell death and survival, nervous system development and function, cellular compromise. **b** Interaction network of genes harboring rare polymorphisms detected in homozygosis in the patient (network score = 12) is associated with the top disease and functions: cell cycle, digestive system development and function, hair and skin development and function. **c** Interaction network of genes affected by compound heterozygous variants (network score = 25) is associated with the top disease and functions: cancer, organismal injury, abnormalities, and gastrointestinal disease. Continuous and dashed lines indicate direct and indirect interactions between molecules, respectively. Blue molecules represent the genes encountered in our analysis and blank molecules represent other genes automatically included by IPA. Molecules are displayed by various shapes depending on the functional class of the gene product, according to IPA Path designer shapes (Additional file 7)

Gene ontology enrichment analysis of the 12 genes harboring protein-affecting somatic mutations revealed several biological processes such as muscle tissue/organ development (*ZFPM2* and *MEGF10*), which is related to the mesothelium origin of this tumor, cell adhesion (*DPP4*, *CDH9*, *CNTNAP4*, *MEGF10*), response to mechanic stimulus (*MEIS2*, *TRPA4*), and response to abiotic stimulus (*DPP4*, *MEIS2*, *TRPA1*) (Additional file 3: Table S2). Intriguingly, biological network analysis obtained using Ingenuity Pathway Analysis (IPA) interconnected the 15 genes harboring somatic mutations in a single network associated with cell death and survival, cell damage or degeneration, and nervous system development and function (Fig. 1a).

### Searching for germline variants associated with DSRCT

In an attempt to identify de novo germline mutations and inherited variants potentially associated with DSRCT, we performed whole-exome sequencing of the leukocyte DNA from the patient’s parents (Additional file 2: Table S1). For selecting genetic variants, a minimum coverage of 10× in all samples with at least 30% frequency of the variant allele was considered. Based on these criteria, no de novo variants were found.

Next, we explored the possibility of finding genetic variants associated with DSRCT in an autosomal recessive model of inheritance. First, we searched for polymorphisms (MAF ≤10%) occurring in homozygosity in the patient that were inherited from both heterozygous parents. Four polymorphisms inherited in homozygosis were found in *ADAMTS12*, *RASSF1*, *VEZT*, and *ISX* genes (population frequency ranging from 2.0 to 7.8%). All polymorphisms lead to missense alterations, two of them reported as possibly disease associated by at least one pathogenicity prediction program (Table 2). Interestingly, biological network analysis showed an interconnection between the four genes in a single network from IPA, showing association with cell cycle and digestive system development and function (Fig. 1b).

**Table 2:** Description of rare polymorphisms detected in homozygosity in the DSRCT patient. All variants were validated by sanger sequencing

Gene symbol	cDNA change	Protein change	Type	dbSNP (MAF)	Patient (Frequency/ Coverage)	Mother (Frequency/ Coverage)	Father (Frequency/ Coverage)	Polyphen	Sift	Mutation Taster
*VEZT*	c.1486G > A	p.V496I	Missense	rs10507051 (0.0302)	100% / 17	17.9% / 28	35.0% / 80	PD	T	DC
*ISX*	c.248G > A	p.R83Q	Missense	rs8140287 (0.0308)	100% / 13	37.5% / 24	50.0% / 70	PrD	T	DC
*RASSF1*	c.409G > T	p.A137S	Missense	rs2073498 (0.0711)	83.3% / 12	27.5% / 40	53.1% / 32	B	T	P
*ADAMTS12*	c.3529 T > C	p.W1177R	Missense	rs3813474 (0.0513)	100% / 51	42.0% / 88	45.0% / 40	B	T	P

*MAF* Minor allele frequency, *PrD* Probably damaging, *B* Benign, *PD* Possibly damaging, *D* Damaging, *T* Tolerated, *DC* Disease causing, *P* Polymorphism. (*) low confidence prediction

Next, to identify candidate genes affected by compound heterozygosity, we looked for genes containing two distinct variants inherited independently from each parent. We could confirm 11 genes affected by germline compound heterozygous mutations, in which one variant allele was inherited from the mother and the other variant allele was inherited from the father (MAF ≤10%) (Table 3). We speculate that these genes could be associated with risk to DSRCT development in an autosomal recessive model of inheritance.

**Table 3:** Description of compound heterozygous variants detected in the DSRCT patient. Each one of the variants was exclusively inherited by one of the parents. The genotype and variant frequency were obtained by leukocyte DNA sequencing. All variants were validated by Sanger sequencing

Gene	cDNA change	Protein change	dbSNP (MAF)	Mother		Father		Patient		Polyphen	Sift	Mutation Taster
				Genotype	Variant Frequency	Genotype	Variant Frequency	Genotype	Variant Frequency			
*C2CD3*	c.5653 T > C	p.S1885P	rs142277857 (0.001)	T/C	43.1	T/T	-	T/C	53.57	PrD	D	P
	c.3223A > C	p.S1075R	*-*	A/A	-	A/C	50.91	A/C	41.67	PrD	D	DC
*FAM13C*	c.1361G > A	p.R454H	rs369226393	G/A	56.4	G/G	-	G/A	50	B	T	P
	c.439C > T	p.P147S	rs73299227 (0.0092)	C/C	-	C/T	51,43	C/T	36.36	PD	D*	DC
*GOLGA3*	c.209G > A	p.G70E	rs2291256 (0.0581)	G/A	52.3	G/G	-	G/A	47.06	B	D*	P
	c.3728G > A	p.R1243Q	rs140646528 (0.0134)	G/G	-	G/A	60	G/A	25	B	T	P
*LAMB2*	c.1424G > A	p.R475Q	rs370565848	G/A	36.4	G/G	-	G/A	60	PrD	T	P
	c.5293G > A	p.A1765T	rs74951356 (0.0130)	G/G	-	G/A	55.81	G/A	41.18	B	T	DC
*MTMR6*	c.685C > G	p.P229A	rs149526134 (0.0002)	C/G	60.9	C/C	-	C/G	26.32	B	T	P
	c.1795G > A	p.A599T	rs62619824 (0.0571	G/G	-	G/A	35.09	G/A	51.85	B	T	DC
*RSPH1*	c.742G > A	p.G248R	rs117385282 (0.0839)	G/A	50	G/G	-	G/A	31.25	B	T	P
	c.733G > A	p.G245R	rs151158140 (0.0026)	G/G	-	G/A	50.77	G/A	58.33	PD	T	P
*SLC9A9*	c.1765A > G	p.I589V	rs2289491 (0.0290)	A/G	31.1	A/A	-	A/G	28	B	T	P
	c.1618A > G	p.I540V	rs16853300 (0.0066)	A/A	-	A/G	48.62	A/G	36.36	B	T	P
*SPICE1*	c.2470A > C	p.T824P	rs57006145 (0.0313)	A/C	40.4	A/A	-	A/C	62.16	PrD	T	DC
	c.850G > A	p.V284M	rs73239152 (0.0078)	G/G	-	G/A	47.54	G/A	29.27	B	D	DC
*SYNE1*	c.16277C > T	p.T5426M	rs2306914 (0.0463)	C/T	39.1	C/C	-	C/T	41.07	B	T	P
	c. 12442G > C	p.D4148H	rs117501809 (0.0124)	G/G	-	G/C	49.15	G/C	28.57	PrD	D	P
*TAF5L*	c.721G > A	p.V241I	rs55655740 (0.0042)	G/A	36.0	G/G	-	G/A	48	B	-	DC
	c.1123A > G	p.T375A	rs41304137 (0.0008)	A/A	-	A/G	20	A/G	40	PrD	T	P
*TTN*	c.106619 T > C	p.I35540T	rs55880440 (0.0046)	T/C	40.0	T/T	-	T/C	57.69	-	-	P
	c.65147C > T	p.S21716L	rs13021201 (0.0108)	C/C	-	C/T	42.86	C/T	58.14	-	-	P

*MAF* Minor allele frequency, *PrD* Probably damaging, *B* Benign, *PD* Possibly damaging, *D* Damaging, *T* Tolerated, *DC* Disease causing, *P* Polymorphism. (*) low confidence prediction

Gene ontology analysis of these 11 genes showed enrichment of biological processes related to muscle tissue development (*LAMB2*, *SYNE1*, and *TTN*), morphogenesis (*C2CD3* and *TTN*), and cell cycle (*RSPH1*, *TTN*, and *SPICE1*) (Additional file 4: Table S4). Functional analysis of IPA revealed that 9 of the 11 genes are interconnected in a single network related to cancer, organismal injury and abnormalities, and gastrointestinal disease.

### Copy number alterations

**Fig. 2:**
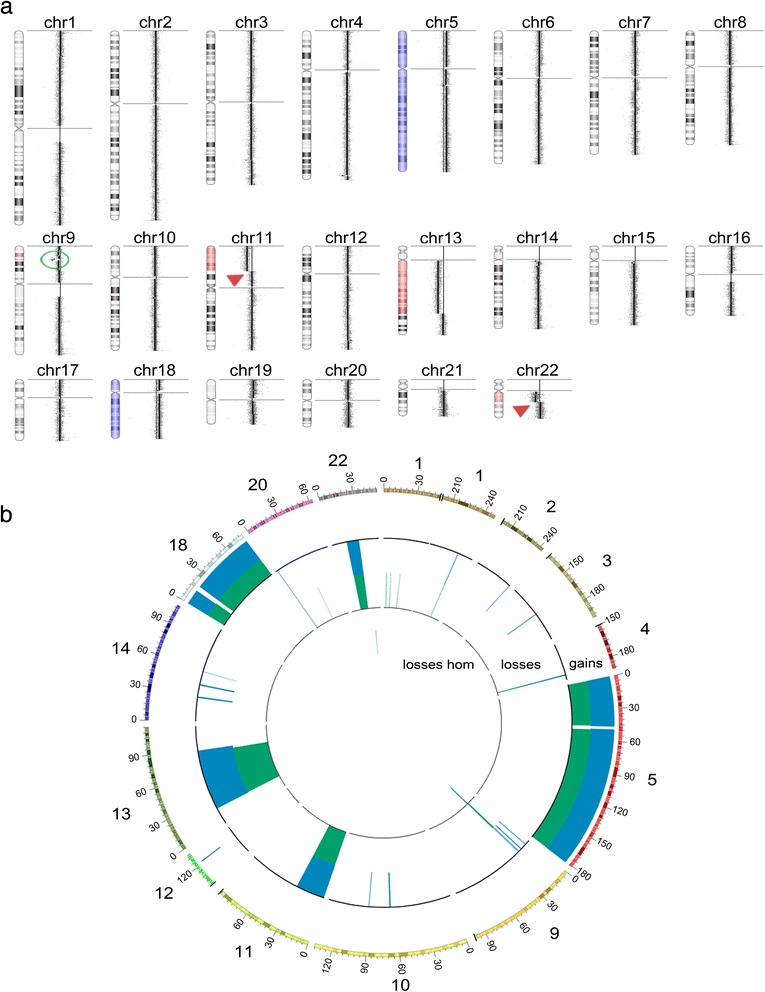
Array CGH profile showing the pattern of somatic copy number alterations detected in the DSRCT genome. **a** Copy number alterations detected by array CGH analysis using a 180-K platform with an effective resolution of ~70 Kb: aneuploidy of chromosomes 5 and 18 (gains, *in blue*), and partial losses of chromosome 13q, 11p, and 22q (*in red*). The *green circle* indicates the focal deletion of a segment of 1.3 Mb at 9p24.1. *Arrows* indicate chromosome 11 and chromosome 22 breakpoints, 11p13 and 22q12.2, respectively. **b** *Circus* plot shows the copy number alterations detected by array CGH and WES. Only the genomic regions affected by CNA events are represented. The numbers on each chromosome region are described in megabases. In *blue*, data from array CGH and in *green* data from WES. A great overlap of CNA detection can be observed using both approaches

Genomic copy number alterations (CNA) were investigated in the DSRCT tumor sample using array CGH in a 180-K platform. Few copy number alterations were detected (Fig. 2a), including aneuploidies such as gain of chromosomes 5 and 18, and 11p, 13q, and 22q deletions. Only one small focal homozygous deletion was identified in a segment of ~1.3 Mb at 9p22.2 (chr9:17,106,384-18,449,088; hg19), encompassing the *CNTLN* and *SH3GL2* genes.

The detection of copy number losses affecting terminal segments of 11p and 22q suggested the presence of the chromosomal translocation t(11;22)(p13;q12) involving *EWSR1* and *WT1* genes.

Additionally, we used NGS data from the WES of the tumor and patient’s leukocyte to search for CNAs, and 98.4% of the events detected by array CGH were validated, demonstrating the efficacy of WES for identification of a wide range of somatic events, besides point mutations and indels (Fig. 2b; Additional file 5: Table S3).

### Establishment of a personalized monitoring strategy based on detection of circulating tumor DNA (ctDNA)

**Fig. 3:**
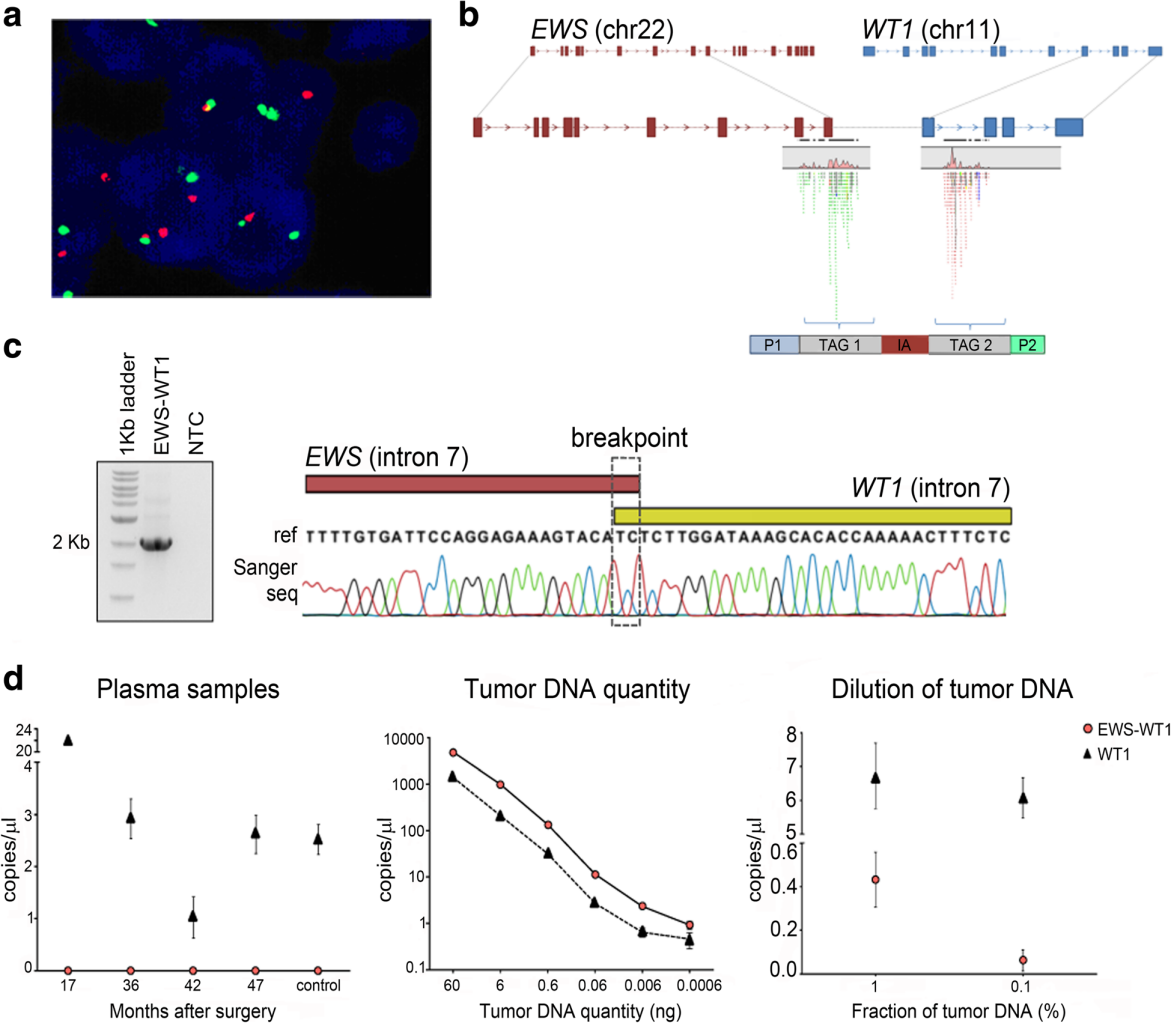
Use of the chromosomal translocation t(11;22)(p13;q12) as a personalized tool for patient monitoring along follow-up. **a** FISH analysis shows break apart probes for *WT1* gene, indicating the occurrence of the fusion. **b** Mate-pair whole-genome sequencing detected paired reads mapping to *EWS* and *WT1* genes. **c** PCR amplification followed by Sanger sequencing confirmed the breakpoint region involving intronic regions of *EWS* and *WT1* genes. **d** Digital droplet PCR assays for detection of the somatic rearrangement EWS-WT1. *Left panel*—Screening of ctDNA from plasma samples collected serially along patient follow-up by ddPCR. No gene fusion was detected in ctDNA from the patient collected in four different time points after surgery, suggesting no relapse, recurrence, or progression of the disease. Presence of cell-free DNA is shown by detection of non-rearranged WT1 probes in the plasma samples from the patient and from control plasma sample. *Middle panel*—Serial dilutions of tumor DNA to check the sensibility of the approach in detecting the fusion event, starting from 60 ng of input following five dilution series of tenfold as indicated. *Right panel*—Detection of somatic rearrangement in different tumor DNA fractions, 1.0 and 0.1%

The genomic translocation t(11;22)(p13;q12), which is considered the molecular hallmark of this tumor type, was initially detected by FISH analysis for diagnostic purposes. Furthermore, the same translocation was also detected in the array CGH and confirmed by whole-genome sequencing. The mate-pair approach used for whole-genome sequencing followed by validation by PCR and Sanger sequencing allowed the precise delimitation of the chromosomal breakpoints (Fig. 3). This somatic fusion event was then used as a tumor biomarker for monitoring the patient along the follow-up period using a liquid biopsy-based approach. We searched for traces of circulating tumor DNA in plasma samples collected 17, 36, 42, and 47 months after surgery. No signs of the translocation in any of the post-treatment plasma samples were detected (Fig. 3) using digital droplet PCR (ddPCR). The absence of the biomarker in any of the post-treatment plasma samples is in agreement with favorable clinical response of the patient, showing long-term disease-free survival and no sign of disease recurrence. As a positive control, we confirmed the presence of cell-free circulating DNA by detecting non-rearranged *WT1* gene in all plasma samples.

Despite the unavailability of plasma samples at the moment of diagnosis and before treatment (neoadjuvant/adjuvant chemotherapy and surgery) to be used as a baseline positive control, we carried out stringent control assays. We tested the robustness of the approach by evaluating six different amounts of tumor DNA, ranging from 60 ng to 6 pg, and searched for both the tumor-specific fusion event and for the non-rearranged *WT1* gene. We achieved a linear quantification of tumor marker and wild-type DNA, and we were able to discriminate the presence of the fusion event even in extremely low quantity of DNA (6 pg) (Fig. 3d). To test the specificity of the ddPCR assay, we used leukocyte sample of the patient and also DNA from plasma and leukocyte samples of healthy donors, and the absence of detection of tumor-specific fusion confirmed the assay specificity for tumor DNA, without false positive signals (Additional file 6: Figure S2). Further, we mimicked a situation of circulating tumor DNA in body fluid by mixing 1 part of tumor DNA to 100 and 1000 parts of leukocyte DNA, then screened for the fusion event using 1 ng of this DNA mix. Typically, the detection of circulating tumor DNA has been reported as a fraction between 0.1 and 90% of plasma DNA for cancer patients, depending on tumor type and patient characteristics [27]. Here, we were able to detect the fusion event in the 1.0 and 0.1% fractions, supporting the reliability of our established approach for patient surveillance based on liquid-biopsy screening.

## Discussion

DSRCT is an aggressive tumor not yet broadly investigated with the powerful genomic tools available. Mainly due to the rarity of this disease, only a few studies investigated molecular alterations in this tumor type. Shukla et al. [8] screened the occurrence of 275 COSMIC mutations in 29 oncogenes and found no alterations in any of the 24 DSRCT samples investigated. More recently, a study based on targeted exome sequencing of six adult patients with pediatric-type malignancies found *AURKB* and *MCL1* amplifications and variants of unknown clinical significance in *ARID1A* and *RUNX1* genes in one DSRCT [6].

Here, by performing a comprehensive screening of the genomic alterations in a family based approach, we identified somatic and germline variants possibly associated with DSRCT. In our study, the use of a commercially available gene panel did not show to be an adequate strategy. Based on this finding, one can argue that screening by commercially available gene panels is not an effective approach for most cases of rare tumors, since the targeted genes represented in these panels are usually those well characterized in common solid tumors. On the other hand, the use of WES not only revealed mutated genes but also showed robustness for detecting DNA copy number alterations. Concordance rates between WES and array CGH (the gold standard for CNA screening) were above 98% (Fig. 2). Among the CNAs not detected in the WES analysis, two of them were mapped to regions not covered by the library probes and five were low-level mosaic alterations (Additional file 3: Table S2). Thus, if we consider CNAs mapping to WES target regions and non-mosaic CNAs, concordance rates were above 99.8%.

In total, 38 somatically acquired alterations, including point mutations (15) and CNAs (23), were detected. The small number of somatic alterations identified here is in agreement with what is expected for pediatric tumors [28]. Moreover, given the occurrence of the driver EWS-WT1 fusion protein, additional oncogenic mutations for tumor onset is probably less necessary.

We identified 12 genes affected by somatic mutations possibly involved with the disease. These genes are involved with cellular development and morphology that are pathways in which *WT1* gene plays an important role. Similarly, the genes affected by compound heterozygous mutations showed enrichment in biological processes of muscle tissue development and morphogenesis. Altogether, these data suggests that disruption of the embryonic cellular development process is involved with DSRCT onset, which is commonly seen in pediatric tumors.

Another interesting finding is that among the set of somatically mutated genes, 8 showed to be mutated in desmoplastic melanoma samples from TCGA project [29, 30], in frequencies ranging from 5 to 25% of the 20 samples interrogated. This data suggests that mutation in these genes might be involved with the desmoplastic phenotype, seen in both tumor types.

Additionally, 7 out of 15 genes harboring somatic mutations (*CHL1*, *MEGF10*, *MEIS2*, *MYH8*, *RIMS4*, *TBPL1*, and *ZFPM2*) are regulated by the same transcription factor, LEF1 (*p* < 0.001 by enrichment analysis), which, in turn, is regulated by WT1 [31]. We therefore postulate that DSRCT tumors presenting increased activity of WT1 due to EWS-WT1 fusion might upregulate the expression of several genes mediated by LEF1 transcription factor. However, the accumulation of mutations in this set of genes regulated by LEF1 activation and its interrelation with the EWS-WT1 fusion protein remains to be addressed.

Finally, the definition of the precise genomic breakpoint of the t(11;22)(p13;q12) translocation by whole-genome and Sanger sequencing enabled the development of a personalized tool to precisely monitor the presence of ctDNA in plasma samples during the patient’s follow-up. Detection of tumor-specific genomic rearrangements has been shown as a sensitive and specific method for monitoring of disease status of cancer patients [32–35] and has clear advantages over point mutations concerning the specificity of detection [33]. Here, we applied ctDNA screening in plasma samples collected in three clinical appointments during 3 years after surgery and, up to now, we did not detect the presence of this tumor marker. These results are in agreement with imaging exams (CT scan and PET scan) showing no signs of disease and also with good overall clinical condition of the patient and highlight the applicability of using genomic rearrangement for building personalized tool for patient surveillance. After defining the genomic breakpoint of EWS-WT1 fusion, DSRCT patients can benefit from a highly specific test that has the advantage of a rapid turnaround time and potentially higher sensitivity in detecting disease progression earlier than imaging exams or other cancer antigens measurements, as reported for other tumor types [36, 37]. Monitoring through liquid biopsy is particularly attractive for solid tumors, which cannot be repeatedly sampled without more invasive procedures. Considering the rarity of this subtype of sarcoma and the lack of effective treatment, the detection of specific tumor marker and the monitoring of its persistence can improve the identification of patients with worse prognosis to tailor the treatment more properly. Thus, the perspective is to employ the approach used here for new patients and improve the outcome for those with worse prognosis.

## Conclusion

To our knowledge, this is the first comprehensive genomic characterization of one DSRCT case. Continuous efforts to establish the genomic landscape of rare diseases, frequently neglected in large sequencing consortiums, are highly significant to improve the knowledge of defective pathways involved with tumor onset in general, in addition to the strong potential of revealing druggable targets for clinical use.

## Additional files

Additional file 1: Figure S1. Patient medical history and sample collection time points. Chemotherapy treatment marked in green consisted of 4 cycles of vincristine, cyclophosphamide, and doxorubicin (VAC) alternated with ifosfamide, carboplatin, and etoposide (ICE). Chemotherapy treatment marked in red consisted of hyperthermic intraperitoneal chemotherapy (HIPEC) with cisplatin and doxorubicin. Abdominal irradiation total of 30 Gy. (JPG 96 kb)
Additional file 2: Table S1. Statistics of sequencing results. Sequence coverage by Comprehensive Cancer Panel (Thermo Scientific), Whole Exome Sequencing (Thermo Scientific) and Whole Genome Sequencing (Mate-Paired approach) (Thermo Scientific). (DOC 38 kb)
Additional file 3: Table S2. Gene Ontology enriched categories of genes affected by somatic mutations. Biological processes with a p-value <0,001 was considered based on Webgestalt annotation tool [38, 39]. (DOC 31 kb)
Additional file 4: Table S4. Gene Ontology-enriched categories of genes affected by compound heterozygous mutations. Biological processes with a *p* value <0.001 was considered based on WebGestalt annotation tool [38, 39]. (DOC 32 kb)
Additional file 5: Figure S3. Copy Number Alterations detected by array CGH and confirmed by WES. (DOC 57 kb)
Additional file 6: Figure S2. Screening of ctDNA in leukocyte samples. Pre-surgery sample collected at day of surgery. Post-surgery sample collected at 22 months after diagnosis (17 months after surgery). (JPG 75 kb)
Additional file 7: Description of the functional class of each molecule shape used by IPA. (PDF 99 mb)

## Abbreviations

CGH: Comparative genomic hybridization
CNA: Copy number alterations
COSMIC: Catalogue of Somatic Mutations in Cancer
CT: Computed tomography
ctDNA: Circulating tumor DNA
ddPCR: Digital droplet PCR
DSRCT: Desmoplastic small round cell tumor
GATK: Genome Analysis Toolkit
HIPEC: Hyperthermic intraperitoneal chemotherapy
ICE: Ifosfamide, carboplatin, and etoposide
Indel: Small insertions and deletions
IPA: Ingenuity pathway analysis
MAF: Minor allele frequency
PET: Positron emission tomography
SNP: Simple nucleotide polymorphism
TCGA: The cancer genome atlas
VAC: Vincristine, cyclophosphamide, and doxorubicin
WES: Whole-sequencing
