# Supplementary figures and images for: A genomic case study of desmoplastic small round cell tumor: comprehensive analysis reveals insights into potential therapeutic targets and development of a monitoring tool for a rare and aggressive disease
Source: Hum Genomics. 2016 Nov 18;10:36. doi: 10.1186/s40246-016-0092-0 (PMC5116179; doi:10.1186/s40246-016-0092-0)

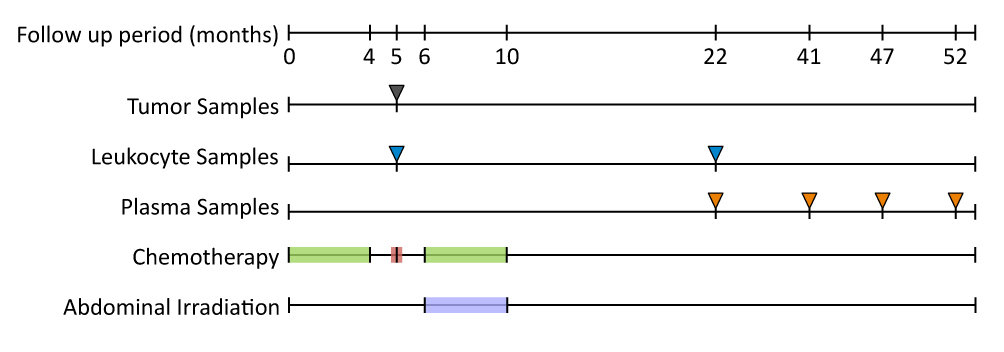

Supplement: Additional file 1: Figure S1. — Patient medical history and sample collection time points. Chemotherapy treatment marked in green consisted of 4 cycles of vincristine, cyclophosphamide, and doxorubicin (VAC) alternated with ifosfamide, carboplatin, and etoposide (ICE). Chemotherapy treatment marked in red consisted of hyperthermic intraperitoneal chemotherapy (HIPEC) with cisplatin and doxorubicin. Abdominal irradiation total of 30 Gy. (JPG 96 kb) [file 40246_2016_92_MOESM1_ESM.jpg]

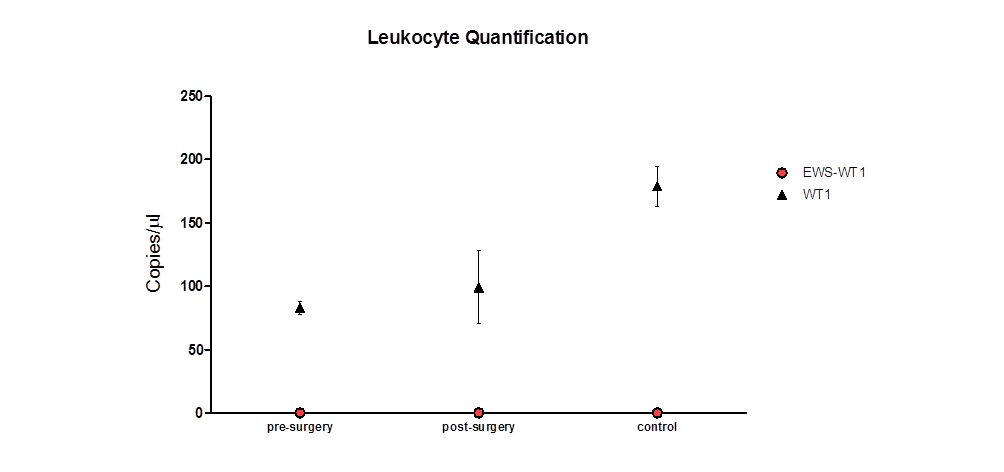

Supplement: Additional file 6: Figure S2. — Screening of ctDNA in leukocyte samples. Pre-surgery sample collected at day of surgery. Post-surgery sample collected at 22 months after diagnosis (17 months after surgery). (JPG 75 kb) [file 40246_2016_92_MOESM6_ESM.jpg]
