# Supplementary material for: A genomic case study of desmoplastic small round cell tumor: comprehensive analysis reveals insights into potential therapeutic targets and development of a monitoring tool for a rare and aggressive disease
Source: Hum Genomics. 2016 Nov 18;10:36. doi: 10.1186/s40246-016-0092-0 (PMC5116179; doi:10.1186/s40246-016-0092-0)
Supplement: Additional file 2: Table S1. — Statistics of sequencing results. Sequence coverage by Comprehensive Cancer Panel (Thermo Scientific), Whole Exome Sequencing (Thermo Scientific) and Whole Genome Sequencing (Mate-Paired approach) (Thermo Scientific). (DOC 38 kb) [file 40246_2016_92_MOESM2_ESM.doc]

**Supplementary Table 1**. Statistics of sequencing results. Sequence coverage by Comprehensive Cancer Panel (Thermo Scientific), Whole Exome Sequencing (Thermo Scientific) and Whole Genome Sequencing (Mate-Paired approach) (Thermo Scientific).

|  | Tumor | Blood | Mother | Father |
| --- | --- | --- | --- | --- |
| **Comprehensive Cancer Panel** |  |  |  |  |
| Number of reads mapped on target | 477,394,521 |  |  |  |
| Percentage of reads mapped to target regions | 95.90% |  |  |  |
| Mean Coverage | 282.7X |  |  |  |
| Percentage of reads with min.10X coverage | 80.3% |  |  |  |
| **Whole Exome Sequencing** |  |  |  |  |
| Number of bases mapped on target | 28 Mb | 33 Mb | 2.7 Mb | 0.9 Mb |
| Percentage of reads with min.20X coverage | 63% | 67% | 40% | 17% |
| **Whole Genome Sequencing (Mate-pair)** |  |  |  |  |
| Total number of bases | 26 Gb | 13 Gb |  |  |
| Mean Coverage | 8.45 X | 4.19 X |  |  |
