# Supplementary material for: A genomic case study of desmoplastic small round cell tumor: comprehensive analysis reveals insights into potential therapeutic targets and development of a monitoring tool for a rare and aggressive disease
Source: Hum Genomics. 2016 Nov 18;10:36. doi: 10.1186/s40246-016-0092-0 (PMC5116179; doi:10.1186/s40246-016-0092-0)
Supplement: Additional file 3: Table S2. — Gene Ontology enriched categories of genes affected by somatic mutations. Biological processes with a p-value <0,001 was considered based on Webgestalt annotation tool [38, 39]. (DOC 31 kb) [file 40246_2016_92_MOESM3_ESM.doc]

**Supplementary Table 2.** Gene Ontology enriched categories of genes affected by somatic mutations. Biological processes with a p-value<0,001 was considered based on Webgestalt annotation tool [38, 39].

| Biological Process | P-value | Genes |
| --- | --- | --- |
| Cell-cell adhesion | P=0.0061 | *DPP4, CDH9, MEGF10* |
| Biological adhesion | P=0.0014 | *DPP4, CDH9, CHL1, CNTNAP4, MEGF10* |
| Cell adhesion | P=0.0014 | *DPP4, CDH9, CHL1, CNTNAP4, MEGF10* |
| Response to mechanical stimulus | P=0.0071 | *MEIS2, TRPA1* |
| Muscle tissue development | P=0.0346 | *ZFPM2, MEGF10* |
| Muscle organ development | P=0.0408 | *ZFPM2, MEGF10* |
| Response to abiotic stimulus | P=0.0386 | *DPP4, MEIS2, TRPA1* |
| Striated muscle tissue development | P=0.0322 | *ZFPM2, MEGF10* |
