# Supplementary material for: A genomic case study of desmoplastic small round cell tumor: comprehensive analysis reveals insights into potential therapeutic targets and development of a monitoring tool for a rare and aggressive disease
Source: Hum Genomics. 2016 Nov 18;10:36. doi: 10.1186/s40246-016-0092-0 (PMC5116179; doi:10.1186/s40246-016-0092-0)
Supplement: Additional file 4: Table S4. — Gene Ontology-enriched categories of genes affected by compound heterozygous mutations. Biological processes with a p value <0.001 was considered based on WebGestalt annotation tool [38, 39]. (DOC 32 kb) [file 40246_2016_92_MOESM4_ESM.doc]

**Supplementary Table 4**. Gene Ontology enriched categories of genes affected compound heterozygous mutations. Biological processes with a p-value<0,001 was considered based on Webgestalt annotation tool [38, 39].

| Biological Process | P-value | Genes |
| --- | --- | --- |
| Striated muscle cell development | P=0.0029 | *LAMB2, TTN* |
| Muscle fiber development | P=0.0017 | *LAMB2, TTN* |
| Muscle cell differentiation | P=0.0007 | *LAMB2, SYNE1, TTN* |
| Muscle structure development | P=0.0030 | *LAMB2, SYNE1, TTN* |
| Muscle cell development | P=0.0042 | *LAMB2, TTN* |
| Striated muscle cell differentiation | P=0.0081 | *LAMB2, TTN* |
| Chromosome segregation | P=0.0042 | *TTN, SPICE1* |
| M phase | P=0.0049 | *RSPH1, TTN, SPICE1* |
| Cellular component assembly involved in morphogenesis | P=0.0037 | *C2CD3, TTN* |
| Heart morphogenesis | P=0.0058 | *C2CD3, TTN* |
