# Supplementary material for: A genomic case study of desmoplastic small round cell tumor: comprehensive analysis reveals insights into potential therapeutic targets and development of a monitoring tool for a rare and aggressive disease
Source: Hum Genomics. 2016 Nov 18;10:36. doi: 10.1186/s40246-016-0092-0 (PMC5116179; doi:10.1186/s40246-016-0092-0)
Supplement: Additional file 5: Figure S3. — Copy Number Alterations detected by array CGH and confirmed by WES. (DOC 57 kb) [file 40246_2016_92_MOESM5_ESM.doc]

**Supplementary Table 3**. Copy Number Alterations detected by array CGH and confirmed by WES.

| **Chromossome** | **Cytoband** | **Genomic Coordinates (Build 37)** | **Event** | **Segment size (pb)** | **WES** |
| --- | --- | --- | --- | --- | --- |
| chr1 | q42.2 | chr1:232,232,138-232,811,281 | CN Loss | 579.144 | CN Loss confirmed |
| chr3 | q26.1 | chr3:165,221,640-165,720,937 | CN Loss | 499.298 | not detected |
| chr4 | q35.1 | chr4:185,109,067-186,221,396 | CN Loss | 1.112.330 | CN Loss confirmed |
| chr5 | p15.33 - p11 | chr5:0-46,365,277 | CN Gain | 46.365.278 | CN Gain confirmed |
| chr5 | q11.1 - q35.3 | chr5:49,083,933-180,915,260 | CN Gain | 131.831.328 | CN Gain confirmed |
| chr9 | p24.1 | chr9:4,806,268-5,336,080 | CN Loss^#^ | 529.813 | not detected |
| chr9 | p23 | chr9:11,680,361-12,557,437 | CN Loss^#^ | 877.077 | not covered |
| chr9 | p22.3 | chr9:15,589,631-16,108,786 | CN Loss | 519.156 | CN Loss confirmed |
| chr9 | p22.2 | chr9:16,803,294-17,106,384 | CN Loss | 303.091 | CN Loss confirmed |
| chr9 | p22.2 | chr9:17,106,384-18,449,088 | Homozygous Copy Loss | 1.342.705 | CN Loss confirmed* |
| chr10 | q21.3 | chr10:66,619,838-67,854,353 | CN Loss^#^ | 1.234.516 | not detected |
| chr10 | q23.31 | chr10:90,435,299-90,835,511 | CN Loss^#^ | 400.213 | not detected |
| chr11 | p15.5 - p13 | chr11:0-32,416,083 | CN Loss | 32.416.084 | CN Loss confirmed |
| chr12 | q24.31 | chr12:121,800,732-122,486,667 | CN Gain^#^ | 685.936 | not detected |
| chr13 | q11 - q31.1 | chr13:19,024,748-87,395,415 | CN Loss | 68.370.668 | CN Loss confirmed |
| chr14 | q12 | chr14:26,257,714-27,554,207 | CN Loss^#^ | 1.296.494 | not detected |
| chr14 | q21.1 | chr14:40,280,929-41,708,660 | CN Loss^#^ | 1.427.732 | not covered |
| chr14 | q22.3 | chr14:56,106,297-56,237,089 | CN Loss | 130.793 | not detected |
| chr18 | p11.32 - p11.21 | chr18:0-14,074,683 | CN Gain | 14.074.684 | CN Gain confirmed |
| chr18 | q11.1 - q23 | chr18:18,529,851-78,077,248 | CN Gain | 59.547.398 | CN Gain confirmed |
| chr20 | p13 | chr20:1,353,139-1,706,869 | CN Loss | 353.731 | CN Loss confirmed |
| chr22 | q11.1 - q11.21 | chr22:16,061,206-19,698,074 | CN Loss | 3.636.869 | CN Loss confirmed |
| chr22 | q11.21 - q12.2 | chr22:19,811,430-29,684,118 | CN Loss | 9.872.689 | CN Loss confirmed |

Not Covered: regions not covered by the WES capture probes; Not detected: no CNA event detected by WES; * region partially covered by WES; ^#^ copy number alterations in mosaic.
