# Supplementary material for: A genomic case study of desmoplastic small round cell tumor: comprehensive analysis reveals insights into potential therapeutic targets and development of a monitoring tool for a rare and aggressive disease
Source: Hum Genomics. 2016 Nov 18;10:36. doi: 10.1186/s40246-016-0092-0 (PMC5116179; doi:10.1186/s40246-016-0092-0)
Supplement: Additional file 7: — Description of the functional class of each molecule shape used by IPA. (PDF 99 mb) [file 40246_2016_92_MOESM7_ESM.pdf]

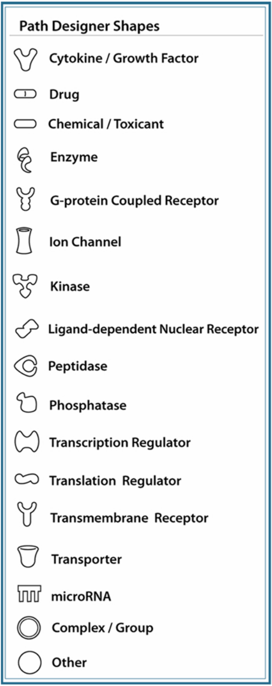
Path Designer Shapes
Cytokine / Growth Factor
Drug
Chemical / Toxicant
Enzyme
G-protein Coupled Receptor
Ion Channel
Kinase
Ligand-dependent Nuclear Receptor
Peptidase
Phosphatase
Transcription Regulator
Translation Regulator
Transmembrane Receptor
Transporter
microRNA
Complex / Group
Other
